# Crossing the metabolic homeostasis divide: panoramic decoding of therapeutic targets for metabolic-inflammatory crosstalk in rheumatoid arthritis

**DOI:** 10.3389/fimmu.2025.1633752

**Published:** 2025-09-11

**Authors:** Siyu Liang, Lei Wan, Siyu Wang, Mengyu Zhang, Ying Wang, Wenwen Min, Yu Zhang

**Affiliations:** 1 The First Affiliated Hospital of Anhui University of Chinese Medicine, Anhui, Hefei, China; 2 Anhui University of Chinese Medicine First Clinical Medical College, Anhui, Hefei, China

**Keywords:** rheumatoid arthritis, glucose metabolism, lipid metabolism, inflammations, immunity, target of intervention

## Abstract

Rheumatoid arthritis (RA) is an autoimmune disease characterized by chronic inflammation and joint destruction. Its pathogenesis is closely related to the imbalance of glycolipid metabolism. This article reviews the pathophysiological mechanisms of glycolipid metabolism in the RA pathogenesis, focusing on the physiological mechanisms of glucose and lipid metabolism as well as the characteristics of glycolipid metabolism imbalance and their interactions in RA. Moreover, this study highlights the relationship between specific glycolipid metabolism markers and disease activity, as well as the innovative targets and intervention strategies of glycolipid metabolism modulation in the RA treatment. Studies show that RA patients have over-activated glycolytic pathways and disrupted lipid metabolism. These metabolic changes drive the inflammatory response and joint destruction and are also strongly associated with disease activity. Through a deeper understanding of the key nodes and regulatory mechanisms of glycolipid metabolism in RA, this article might provide new ideas for the precise diagnosis and treatment of RA.

## 1 Introduction

**Figure 1:**
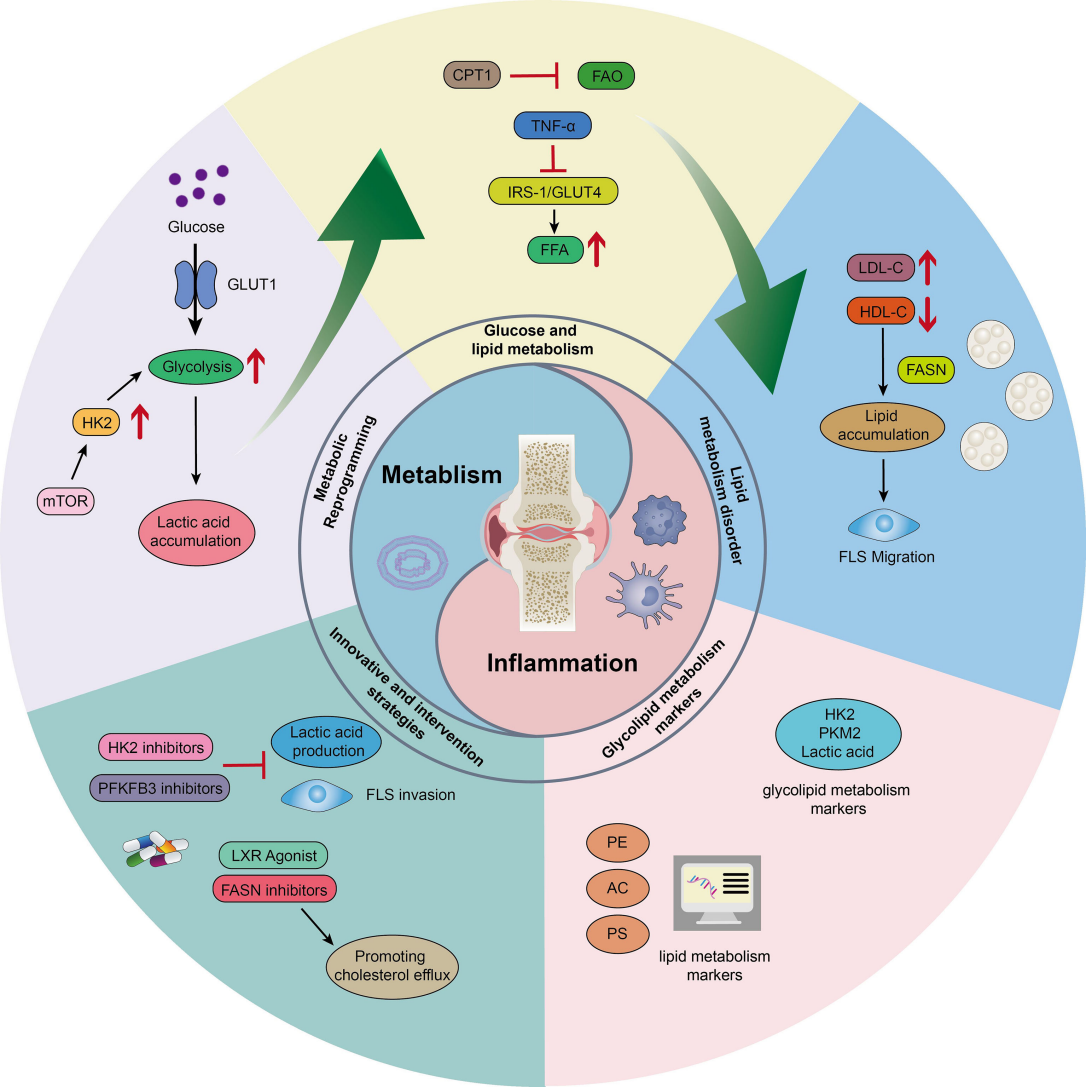
Graphical abstract.

Rheumatoid arthritis (RA) is a systemic autoimmune disease characterized by chronic synovitis and joint destruction. Its global prevalence rate is about 0.5-1% with 2–3 times higher incidence rate in women than that in men, and the total number of patients in China exceeds 5 million (1). The disability rate increases dramatically with the course of the disease, reaching 70% at 3 years in untreated patients, and the systemic complications reduce life expectancy by 10–15 years (2). Currently, there are three core challenges in the management of RA: early joint protection, management of systemic complications, and regulation of metabolic-inflammatory interactions. The RA-related abnormalities of glucose and lipid metabolism are particularly prominent in patients with type 2 diabetes mellitus (T2DM) with a 15-19% prevalence. The abnormalities in lipid metabolism occur in 51-68% of cases, as evidenced by elevated triglycerides (TG) and low-density lipoproteins (LDL-C) levels and low high-density lipoproteins (HDL-C) levels (3, 4). This metabolic disorder is prevalent in rheumatic diseases, such as ankylosing spondylitis and psoriatic arthritis. However, a unique ‘inflammation-metabolism’ vicious circle is formed in RA. Notably, patients with RA combined with T2DM exhibit more pronounced dyslipidemia and glucose metabolism disorders, characterized by greater insulin resistance, elevated baseline inflammation levels, and more severe lipid profile abnormalities. These manifestations not only affect disease activity (as evidenced by increased DAS28 scores) but also significantly alter the response patterns to metabolic interventions. Current research has yet to systematically evaluate the similarities and differences in metabolic pathway reprogramming mechanisms between RA combined with T2DM and RA patients without T2DM, presenting both challenges and opportunities for precision intervention strategies. The chronic inflammation interferes with insulin signaling through cytokines, such as Tumor necrosis factor-α and Interleukin- 6(TNF-α and IL-6), and inhibits adipocyte differentiation, leading to insulin resistance and lipid accumulation (4, 5). Consequently, metabolic reprogramming (such as hyperglycolysis and enhanced lipid synthesis in synovial fibroblasts) drives joint invasion and vascular opacification formation while accelerating the atherosclerotic process (5). The mechanism of glycolipid metabolism imbalance in RA not only reveals the ‘inflammation-metabolism-immunity’ interaction network but also provides directions for the development of therapeutic strategies targeting the key metabolic nodes. Disrupting the pathological cycle by modulating the metabolic microenvironment might be a breakthrough in inhibiting joint destruction and reducing the risk of complications (**Figure 1**).

## 2 Physiological mechanisms of glucose and lipid metabolism

### 2.1 Core pathways of glucose metabolism and their dynamic synergistic networks

**Figure 2:**
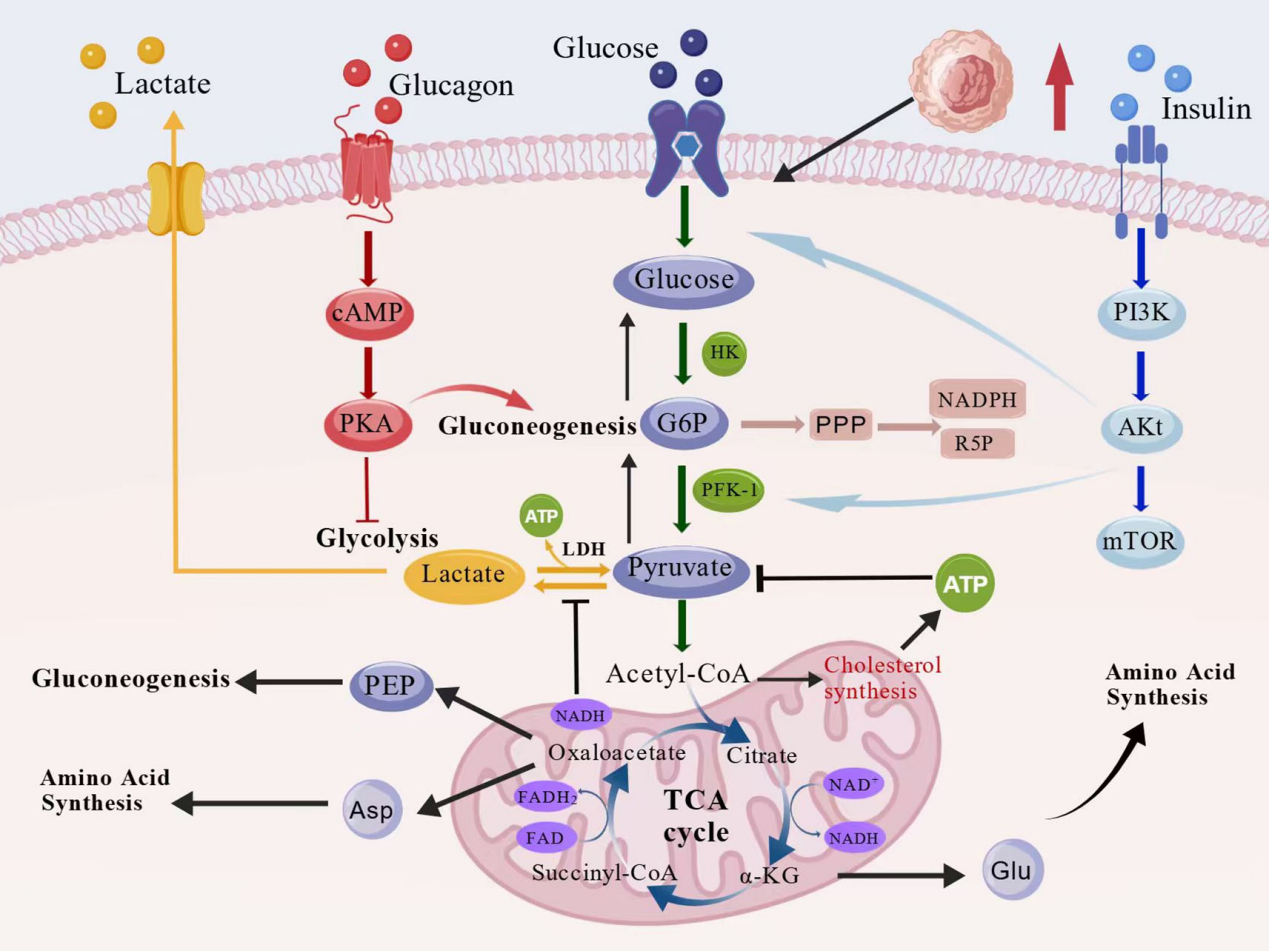
Core pathways of glucose metabolism and their dynamic synergistic networks. Glucose metabolism integrates glycolysis, the tricarboxylic acid (TCA) cycle, and amino acid and lipid synthesis. Insulin activates the PI3K–Akt–mTOR pathway to enhance glucose uptake and glycolysis, whereas glucagon promotes gluconeogenesis through the cAMP–PKA cascade. The TCA cycle acts as a metabolic hub linking carbohydrate, amino acid, and lipid metabolism through acetyl-CoA and key intermediates.

Sugar metabolism is a central mechanism of cellular energy homeostasis. Its core pathways, including glycolysis and the tricarboxylic acid cycle (TCA cycle), achieve energy supply and biosynthesis through synergistic interactions. This synergy is not a simple linear superposition but a spatio-temporal coupling through the dynamic equilibrium of metabolic intermediates. Glycolysis is regulated by key enzymes, such as hexokinase and phosphofructokinase-1 (5, 6). Studies have shown that glycolysis not only provides energy for cells in hypoxic environments, but its product, pyruvate, also enters the mitochondria to participate in the TCA cycle, thus generating a large number of ATPs through oxidative phosphorylation (7). Under hypoxic conditions, pyruvate is preferentially converted to lactate rather than entering mitochondria; this ‘metabolic reprogramming’ phenomenon is pathologically important in anti-inflammation. The TCA cycle acts as a metabolic hub, integrating the breakdown products of sugars, fats and proteins to provide precursors for the synthesis of nucleotides, heme and amino acids (8). Its open characteristics allow the metabolic intermediate to flow into multiple branching pathways (9). This mechanism shows how cells can flexibly regulate energy supply and synthesis needs, and may provide a reference for understanding the origin of abnormal tumor metabolism such as the “Waberger effect” (**Figure 2**).

The glucose metabolic pathway is highly integrated with cellular signaling networks and can be dynamically balanced by metabolite feedback and hormonal regulation. Insulin accelerates glucose uptake and glycolysis by activating the Phosphoinositide 3-kinase-Akt(PI3K/Akt )pathway, while glucagon enhances the activity of key gluconeogenic enzymes through the cAMP-PKA pathway to maintain blood glucose stability (8–10). The tissue specificity in this hormonal regulation provides a new perspective for understanding the mechanisms of insulin resistance in T2DM (11). The TCA cycle plays a crucial role in coordinating the rate of glycolysis with mitochondrial oxidative capacity. This metabolite-mediated negative feedback mechanism achieves ‘pareto optimality’; a balance between energy production and oxidative stress (**Figure 2**).

### 2.2 Dynamic networks and system-level functional regulation of lipid metabolism

Lipid metabolism is a central mechanism, maintaining the energy homeostasis and cellular structure in the organism mainly through fatty acid synthesis and catabolism to achieve energy storage and biofilm construction (12). Fatty acid synthesis can eventually lead to the formation of TGs stored in adipose tissue (12, 13). A study showed that the expression level of fatty acid synthase (FASN) in the liver was positively correlated with lipid accumulation in obese model mice, suggesting its key role in high-fat diet (HFD)-induced metabolic syndrome (14).

In contrast, fatty acid catabolism mainly occurs through the β-oxidation pathway, which breaks down fatty acids into acetyl coenzyme A. The acetyl coenzyme A then enters the TCA cycle, generating a large number of ATPs. The rate of β-oxidation is tightly regulated by carnitine palmitoyl-transferase 1 (CPT1); the CPT1 activity can be inhibited by the denaturation of malonyl coenzyme A. This leads to a ‘metabolic braking’ phenomenon that prevents energy waste (15). At the same time, the acetyl coenzyme A produced by β-oxidation can regulate the abundance of intermediates in the TCA cycle, thus balancing energy metabolism and biosynthetic requirements (16). These findings suggest that β-oxidation might be involved in metabolic reprogramming of cell proliferation. In addition, phosphatidylcholine (PC) and phosphatidylethanolamine (PE) are synthesized via the Cytidine diphosphate-choline/ethanolamine pathway, and their polar heads and hydrophobic tails together form lipid bilayers that provide anchoring sites for membrane proteins (17, 18). These findings suggest that the asymmetric distribution of phospholipids acts as a physical barrier as well as affects vesicle transport and cell migration by regulating membrane curvature.

## 3 Physiological mechanisms underlying the imbalance of glycolipid metabolism in RA

### 3.1 Metabolic reprogramming and regulatory imbalance in RA immune cells

Activated T cells in RA are supported by GLUT1/PFK-1 upregulation and the pentose phosphate pathway (PPP) for proliferation and inflammatory activity (19–21). Lactate accumulation not only acidifies the microenvironment to promote synovial invasion, but also enhances the activation of self-reactive T cells through excessive NADPH caused by PPP bias due to decreased PFKFB3 expression (19, 21). B cells show ‘metabolic resilience’ through the synergistic activation of HK2/LDHA and OXPHOS, driving ACPA secretion and inflammatory amplification (22–27). Notably, B-cell metabolites regulate autoimmune responses through epigenetic modifications, suggesting a deep coupling between metabolic and genetic regulation (28).

This metabolic dysregulation is driven by an aberrant mTOR-HIF-1α-AMPK signaling axis. Briefly, the mTORC1 promotes LDHA/PKM2 expression by activating HIF-1α and catalyzes the reductive carboxylation of glutamine to generate lipid synthesis precursors, thus exacerbating synovial inflammation (26, 29, 30). AMPK inhibition causes impaired fatty acid oxidation and lipid accumulation, while metformin restores metabolic homeostasis by activating AMPK (31–33). At the same time, the synovial hypoxic microenvironment induces neovascularization and enhanced glycolysis via HIF-1α, forming a vicious cycle of inflammation-hypoxia (32).

A study showed that inhibiting PFKFB3 could selectively block Th17 metabolism (21), and the inhibition of G6PD or LDHA restored metabolic imbalances in immune cells (23, 30). Both glutaminase inhibitors (CB-839) and FASN inhibitors (TVB-2640) could block pathological lipid metabolism (27, 30), highlighting the targeting potential of key metabolic nodes. Metabolic regulation should be combined with cell specificity, microenvironmental features, and multi-omics dynamic monitoring in order to achieve precision therapy for RA (30, 34, 35) (**Table 1**).

**Table 1:** Key mechanisms of metabolic regulation in RA immune cells.

Characteristics of metabolism	Mechanisms of regulation	Effect of pathology	Potential therapeutic targets
Hyperactive T-cell PPP pathway	PFKFB3 expression is reduced, and NADPH is overproduced	Activation of autoreactive T cells	PFKFB3 inhibitors
B cell glycolysis/OXPHOS synergy	IL-27-mTOR axis is activated, and FASN mediates lipid synthesis	Increased ACPA secretion	FASN inhibitor (TVB-2640)
Reprogramming of glutamine metabolism	mTORC1 mediates reduced carboxylation	Synovial inflammatory lipid precursor generation	Glutaminase inhibitor (CB-839)
Fatty acid oxidation disorders	AMPK inhibition leads to the inactivation of CPT1	Lipid accumulation and metabolic imbalance	AMPK activator (Metformin)

### 3.2 Hyperactivation of glycolytic pathways in RA synoviocytes

The hyperactivation of glycolysis in RA synoviocytes is centered on HK2, whose mitochondrial-bound state drives pathological processes through the dual role of catalyzing glucose phosphorylation and regulating apoptosis (30, 33, 34). The study confirmed that HK2 was specifically and highly expressed in fibroblast-like synovial cells (FLS) and co-localized with invasive phenotype. Silencing the *HK2* gene could significantly inhibit the migration and invasion of FLS as well as lactate production, while its overexpression enhanced its pro-inflammatory function and positively correlated with ‘tumor-like transformation’ characteristics (33, 35). Mechanistically, HK2 inhibited cytochrome c release by binding to VDAC and blocked FLS apoptosis (36, 37); HIF-1α-mediated hypoxic microenvironment further could upregulate HK2 levels, leading to lactate build-up and acidifying the microenvironment, which could activate ASICs channels to exacerbate pain (37).

The HK2-selective inhibitors (such as 3-BrPA) could specifically inhibit FLS invasiveness in animal models without interfering with normal cellular metabolism (38, 39). The mechanism of action involved specific drug binding sites exposed by conformational changes in HK2. In a mouse model, HK2 inhibitors in combination with JAK inhibitors synergistically inhibited FLS metabolic activity and inflammatory signaling in a combined treatment strategy, resulting in a 70% reduction in joint damage (40). This highlighted the need for combination therapy in anti-inflammation and combating joint damage.

### 3.3 Multidimensional effects of lipid metabolism imbalance in RA

Dysregulation of RA lipid metabolism is characterized by elevated LDL-C levels, impaired HDL-C function, and abnormal PCSK9 accumulation, which directly drive inflammation and immune dysregulation. Studies showed that the serum PCSK9 levels were positively correlated with DAS28 and RF, which exacerbated RA progression through a dual mechanism (41, 42). Omega-6 derivatives (PGE2/LTB4) can activate NF-κB to induce IL-6/TNF-α secretion from FLS, while omega-3 fatty acids (EPA/DHA) can inhibit inflammation by substituting for arachidonic acid; clinical trials have shown that 1.8 g/d EPA+DHA could reduce TNF-α levels by 30% (43–45). Simultaneously, the elevated synovial fluid LPA levels enhanced FLS migration via the GPCR-PI3K/Akt axis, while the LPA antagonist Ki16425 inhibited synovial proliferation in an animal model (44).

Statins can reduce iso-prenylated protein synthesis by inhibiting HMG-CoA reductase, inhibit Th17 differentiation, and reduce IL-17 at low doses; however, at high doses, they can impair Treg function (45, 46). LXR agonists show time-dependent effects, that is, short-term activation of ABCA1 promotes cholesterol outflow to inhibit macrophage inflammation, and long-term upregulation of steroid regulatory element-binding protein (SREBP)-1c to enhance fatty acid synthesis and exacerbate lipid accumulation (44, 45, 47). Lipid peroxidation products trigger the production of ACCP antibody by covalently modifying citrullinated proteins, thus creating an epitope diffusion-driven autoimmune cycle (47). This mechanism directly associates oxidative stress with RA-specific autoantigen production, providing a new target for precision intervention in ACPA-positive patients.

The HK2-HIF-1α axis, a pivotal pathway in RA metabolic reprogramming, demonstrates shared activation patterns across multiple cell types. In synovial fluid (FLS), this pathway regulates glycolysis and anti-apoptotic responses, enhancing cellular invasiveness. Within T/B lymphocytes, it synergizes with mTOR and LDHA/PKM2 to upregulate, driving autoimmune activation. The hypoxic microenvironment coordinates the expression of HK2, GLUT1, and LDHA through HIF-1α, establishing an inflammatory-metabolic positive feedback loop. This mechanism permeates both local (synovial) and systemic (immune) pathological processes of RA, serving as the central nexus connecting the triad of “energy metabolism-immune response-tissue destruction”.

## 4 Interaction and regulation of glycolipid metabolism in RA

### 4.1 Interactions between glycolysis and lipid metabolism

The abnormal glucose metabolism in RA causes the imbalance of lipid metabolism through a multidimensional mechanism. The hyperactivation of key enzymes in synovial glycolysis (HK2/LDH) causes lactate accumulation, inhibits CPT1 activity, blocks fatty acid oxidation (FAO), and enhances lipid synthesis (31, 35, 47). A study demonstrated that the upregulation of glycolytic activity could promote lipid synthase (ACC/FASN) level via HIF-1α signaling, while inhibiting the CPT1-mediated FAO, caused intracellular TG/CE accumulation through a mechanism highly similar to the tumor metabolic reprogramming strategy (48).

Pro-inflammatory cytokines (TNF-α/IL-1β/IL-6) form a ‘metabolic-inflammatory’ positive feedback loop. TNF-α can downregulate adipocyte GLUT4 by Inhibit insulin receptor substrate1 (IRS-1) phosphorylation, increase free fatty acid (FFA) levels by 2-3-fold, and activate TLR4/NF-κB pathway in FLS to promote the secretion of Matrix Metallopeptidase 3 (MMP-3), thus accelerating cartilage degradation (48, 49). IL-1β can activate NLRP3 inflammatory vesicles, upregulate cholesterol synthesis genes (*HMGCR*/*LDLR*) through the nuclear translocation of SCAP-SREBP2, and enhance glycolytic enzyme expression via mTORC1, thereby resulting in a metabolic amplification effect (50). IL-6 can upregulate LXRα via JAK2/STAT3 and inhibit ABCA1-mediated cholesterol efflux, while inducing lncRNA RP11 adsorption of miR-33 to enhance SREBP2 activity, forming an epigenetic regulation cascade (51).

miR-21, highly expressed in macrophages, can cause the accumulation of glycolytic intermediates by inhibiting PFK-M, activate DGAT to promote TG synthesis, and hinder FAO through the PTEN/PI3K/Akt pathway, thus creating a ‘metabolic brake’ effect (49, 52). At the same time, TNF-α and IFN-γ synergistically inhibit PPARγ activity, blocking the adipocyte differentiation and promoting lipolysis, thereby forming a lipid-inflammatory vicious circle (53).

### 4.2 Critical nodes of glycolipid metabolism regulation in RA

The co-regulation of glycolipid metabolism in RA relies on the interaction among metabotropic nuclear receptors (PPARγ/LXR), energy sensors (AMPK), and cholesterol synthesis axis (SCAP-SREBP). LXR mediates reverse cholesterol transport by upregulating ABCA1; however, TNF-α can inhibit its expression, leading to macrophage lipid accumulation (54). Similarly, TNF-α could also block the central role of PPARγ in adipose differentiation, suggesting that anti-inflammatory treatment restores metabolic homeostasis (55, 56). The dysfunction of AMPK, the ‘cellular energy switch’, is significant in RA. Rhubarb acid inhibits its nuclear translocation by phosphorylating the Ser372 site of SREBP1c (which is 60% less phosphorylated in RA patients than healthy controls) through AMPK activation, thereby reducing lipid synthesis in the synovium and ameliorating insulin resistance (56, 57). The aberrant activation of the SCAP-SREBP2 axis promotes HMGCR-mediated cholesterol synthesis and positively correlates with DAS28 score (58–60). The high-glycemic environment enhances SCAP binding capacity to SREBP2 via O-GlcNAc glycosylation modification, creating a metabolic-inflammatory positive feedback (61). The AMPK activator in combination with LXR agonist GW3965 can synergistically inhibit lipid accumulation in synovial membranes (59, 60, 62).

The JAK-STAT pathway exacerbates disease by regulating metabolic enzymes as well as inflammatory factors. The phosphorylated STAT3 directly binds to the HK2 promoter to drive glycolysis, and lactate inhibits lipolysis by activating the GPR81 receptor, thereby creating a metabolic-inflammatory vicious cycle (63). HIF-1α upregulates GLUT1/LDHA expression in hypoxic microenvironments and inhibits FAO by targeting the degradation of CPT1A mRNA via miR-27b (63–65). SOCE deficiency reduces mitochondrial FAO capacity by inhibiting PGC-1α/PPARα signaling, while reduced STIM1/Orai1 complex activity exacerbates mitochondrial dysfunction. Moreover, the calcium channel modulator CM-128 ameliorates the dual metabolic and inflammatory phenotypes in animal models (65, 66).

**Figure 3:**
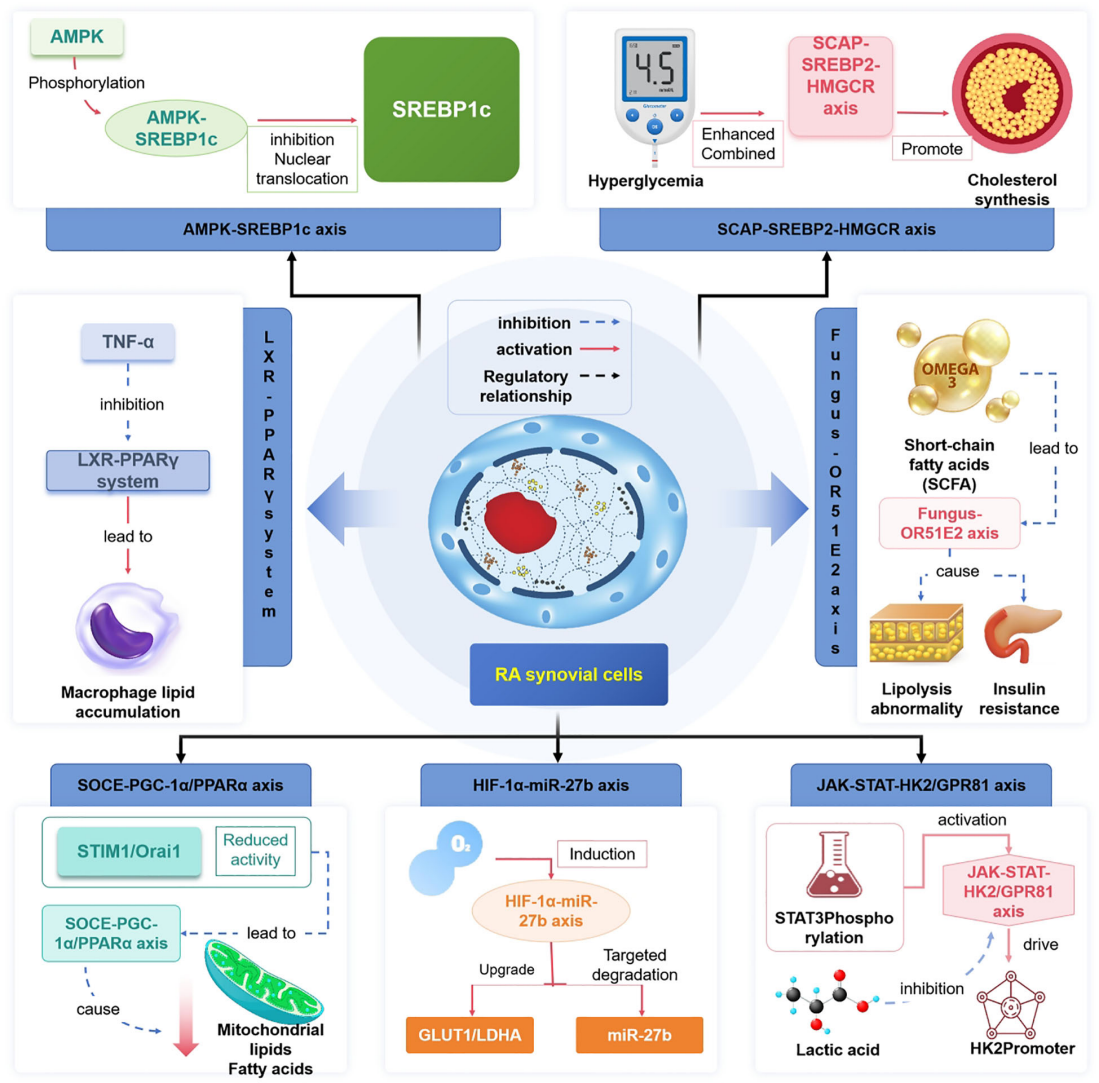
Core regulatory nodes in glycolipid metabolic crosstalk. The left panel illustrates the coordinated activation of HK2/LDHA (glycolysis) by synovial fibroblasts (FLS) and the co-activation of HK2/LDHA+OXPHOS by B cells (driving ACPA secretion). The right panel shows macrophage polarization (M1 pro-inflammatory/M2 anti-inflammatory) regulated by SCAP-SREBP2 (promotes lipid synthesis) and PPARγ (promotes oxidation). Core pathways (HIF-1α↑→ glycolysis, mTOR↑→ proliferation, AMPK↑→ lipid metabolism balance) are differentiated by red/green arrows indicating pro-inflammatory/ anti-inflammatory directions, visually demonstrating the pathological chain of “metabolic reprogramming → immune imbalance → joint destruction”.

The emerging metabolic regulatory nodes include olfactory receptor OR51E2, which is aberrantly expressed in RA adipose tissue, regulates lipolysis through the cAMP-PKA pathway, and is associated with insulin resistance. Short-chain fatty acids (such as acetic acid) are significantly reduced in the gut microbiota of RA patients, leading to OR51E2 ligand deficiency (67–69). HIF-1α inhibitors are indicated in the hypoxic core, and SOCE modulators target the inflammatory spreading zone (70) (**Figure 3**; **Table 2**).

**Table 2:** Key regulatory nodes and intervention strategies of glucose and lipid metabolism in RA.

Node of control	Molecular mechanisms	Effect of pathology
LXR-PPARγ system	TNF-α inhibits LXR/PPARγ activity and blocks cholesterol reverse transport and adipogenesis	Lipid accumulation in macrophages
AMPK-SREBP1c	AMPK phosphorylates SREBP1c at Ser372 and inhibits its nuclear translocation	Increased synovial lipid synthesis/insulin resistance
SCAP-SREBP2-HMGCR axis	High glucose-induced O-GlcNAc modification enhances SCAP-SREBP2 binding	Hypercholesterolemia (associated with DAS28)
JAK-STAT-HK2/GPR81 axis	STAT3 phosphorylation activates the HK2 promoter, and lactate inhibits lipolysis via GPR81	Hyperglycolysis/lipid metabolism disorders
HIF-1α-miR-27b	Hypoxia induces HIF-1α to up-regulate GLUT1/LDHA, and miR-27b targets CPT1A mRNA for degradation	Inhibition of fatty acid oxidation
SOCE-PGC-1α/PPARα	Reduced STIM1/Orai1 activity leads to defective mitochondrial fatty acid oxidation	Mitochondrial dysfunction
gut microbiota-OR51E2 axis	SCFA depletion results in loss of OR51E2 ligand and inhibition of cAMP-PKA signaling	Abnormal lipolysis/insulin resistance

## 5 In-depth exploration of specific glycolipid metabolism markers and disease activity in RA

### 5.1 Association of glycolysis-specific markers with RA disease activity

The abnormal expression of glycolysis-related key enzymes and their metabolites shows a significant correlation with disease activity and progression of bone destruction during the pathological process of RA. Studies showed that the expression levels of HK2, pyruvate kinase type M2 (PKM2), and Lactate dehydrogenase A (LDHA) in synovial tissues of RA patients were 3–5 times higher than those of the healthy population. Moreover, their expression levels were positively correlated with the degree of synovial hyperplasia, serum CRP level, and DAS28 score (68, 69). Notably, PKM2, the terminal rate-limiting enzyme in glycolysis, showed specific high-expression characteristics in activated CD4+ T cells. PKM2 can directly promote Th17 cell differentiation through nuclear translocation. Clinical studies have confirmed a linear positive correlation between serum PKM2 level and Sharp scores, making it a novel biomarker for predicting bone erosion progression (69, 71, 72).

Analyzing the metabolic microenvironmental of synovial fluid revealed a pivotal pathological role for lactate. The 2024 European Congress of Rheumatology (EULAR) guidelines now include synovial fluid lactate concentration >8 mM as a criterion for subclinical inflammation assessment, and its level is significantly and positively correlated with radiographic joint space narrowing scores (73, 74). Lactate can bi-directionally regulate pathological processes via the GPR65 receptor. It can activate the Bcl-2 family anti-apoptotic pathway to maintain synovial cell survival, while it can also induce IL-1β release via NLRP3 inflammatory vesicles, resulting in a 37% increase in the response rate of patients with the high-lactate subtype to IL-1 inhibitor therapy (74–76). In addition, a glycolysis intermediate, Dihydroxyacetone phosphate (DHAP), is specifically enriched in RA synovial fluid and can enhance the ability of FLS pseudopod formation by activating the RhoA/ROCK signaling pathway; preclinical studies showed that the ROCK inhibitor fasudil could inhibit synovial invasion in 40% of cases (77, 78).

The dynamic monitoring of metabolomics revealed characteristic metabolic imbalance patterns in RA. A significant decrease in citrate and α-ketoglutarate in synovial tissue, accompanied by a decrease in the pyruvate/lactate ratio, suggests hyperglycolysis coupled with mitochondrial oxidative metabolism disassociation (75, 78). To quantify the degree of metabolic disorder more intuitively, the researchers proposed a “glycolytic index” (GI), calculated as lactate/pyruvate × α-KG/citrate (78).In brief, this index integrates lactic acid production with TCA cycle inhibition levels to reflect energy metabolism reprogramming trends in inflammatory environments. Demonstrating a significant positive correlation with synovial blood flow signals (r=0.82, p<0.001), it shows potential for non-invasive assessment of RA disease activity. Compared to traditional metabolic parameters, the glycolytic index creates a dynamic indicator reflecting energy metabolism imbalance by integrating glycolytic products and TCA intermediates.

### 5.2 Analysis of the correlation between lipid metabolism-specific markers and disease activity in RA

Multiple specific lipid metabolism markers are present in the serum and synovium of RA patients, and their aberrant expression is closely related to disease progression and inflammatory regulation (76, 78–81).

Membrane phospholipids, A study demonstrated that RA patients exhibited significantly elevated levels of PE (18:1) and lyso-PE (LPE (20:3)), which were positively correlated with Sharp scores (79). PE (18:1) can promote macrophage M1 polarization through the TLR4-MyD88 signaling axis, while LPE (20:3) can activate the synovial fibroblast PI3Kδ pathway, increasing phosphorylation levels by 4.1-fold, thus suggesting its potential value as a combination therapeutic target (79).

Fatty acid classes, Meanwhile, the abnormal accumulation of acylcarnitine (AC (20:3)) in RA serum was associated with mitochondrial FAO defects, and each 1-μM increase in its concentration stimulated a 3-fold increase in synovial IL-1β secretion, thus providing a theoretical basis for metabolic intervention targeting CPT2 (80).

Amino acid derivatives, Furthermore, aspartyl phenylalanine (Asp-Phe) could stabilize HIF-1α by competitively inhibiting prolyl hydroxylase (PHD), forming a metabolic-hypoxic positive feedback loop, while a small-molecule inhibitor targeting its synthetase, Phgdh, could reduce synovial thickness by 42% in a collagen-induced arthritis (CIA) animal model (81, 82).

Lipid metabolism markers are dynamically associated with disease activity. In patients with RA remission, the level of phosphatidyl acid (PA(28:0)) in synovial fluid significantly increased, which stabilized SCAP-SREBP complex on the endoplasmic reticulum by binding to Insig protein, and inhibited the expression of HMGCR, a key enzyme in cholesterol synthesis (76, 78, 79). The abundance of apolipoprotein C-III (ApoC3) in very low density lipoprotein (VLDL) subtype was positively correlated with the rate of joint destruction. The use of antisense oligonucleotide volanesorsen targeting ApoC3 could reduce the bone erosion area in CIA model by 63% (81). In patients with advanced RA, the level of phosphatidylserine (PS 36:1) in synovial fluid increased by 3.2 times, and the secretion of TNF-α was increased by 2.8 times by activating TLR4 pathway, while the inflammation score of experimental arthritis model was reduced by targeting phospholipid flipper Xkr8 (78, 82).

In terms of therapeutic response prediction, specific lipid molecules show great potential as biomarkers. MiR-26a-2-3p inhibits FASN, which reduces palmitic acid synthesis by 72%, thereby reducing the activation level of NLRP3 inflammasome (caspase-1 activity decreased by 54%) (80). The dissociation of PPARα/RXR heterodimers induced by 4-methoxybenzoic acid could be reversed by fenofibrate, and its plasma concentration showed a significant positive correlation with DAS28 score (80). Dynamic monitoring of metabolomics showed that the change of PA (28:0)/PA (32:1) ratio reflected the treatment response two weeks earlier than CRP, providing a new biomarker system for precise adjustment of treatment regimen (76, 79).

The correlation between lipid metabolism-specific markers and RA disease activity was analyzed as follows,

PE (18:1)/LPE (20:3), Promotes the polarization of M1 macrophages through the TLR4-MyD88 axis, and its concentration gradient showed a linear positive correlation with the level of IL-6 in synovium (r=0.72, p <0.001).
AC (20:3), As a biomarker of mitochondrial fatty acid oxidation defect, the level of AC (20:3) increased by 1 μM induced a 3.1-fold increase in IL-1β secretion (p <0.01).
PS (36:1), By activating the TLR4-NFκB signaling pathway, the migration ability of FLS was increased by 4.2 times, and the effect could be inhibited by targeting Xkr8 (p <0.05).
PA (28:0), positively correlated with SREBP2 nuclear translocation (r=0.65, p=0.003), and its level elevation indicates better response to statins.

### 5.3 Complex effects of inflammatory and immune markers on glycolipid metabolism

The pro-inflammatory cytokines TNF-α and IL-6 affect glycolipid metabolic homeostasis in RA patients by targeting the key nodes of metabolism. TNF-α blocks the insulin signaling pathway by inhibiting tyrosine phosphorylation of IRS-1, thereby downregulating GLUT4 expression in adipocytes and elevating HOMA-IR indices; this also causes the inhibition of lipocalin production, resulting in the formation of a pathological cycle of insulin resistance (82–85). Clinical observations showed that the anti-TNF-α therapy reduced the HOMA-IR index by 32%; however, 15% of the patients showed increased LDL-C levels, suggesting the need to optimize the intervention regimen in combination with lipid-modifying therapy. On the other hand, IL-6 promotes synovial RANKL expression and inhibits mitochondrial FAO by activating the JAK2/STAT3 signaling while upregulating lipoprotein lipase activity and accelerating VLDL lipolysis, thus leading to TG metabolism disruption (81, 83). Importantly, IL-6 receptor antagonists can reduce VLDL-TG levels by 28%; however, they might also increase the risk of infection due to inhibition of SAA synthesis, thus requiring strict monitoring of therapeutic windows (83, 86).

IL-1β and IL-10 show antagonistic effects in regulating lipid metabolism. IL-1β promotes the nuclear translocation of the SCAP-SREBP2 complex by activating the NLRP3 inflammatory vesicle and drives HMGCR-mediated cholesterol synthesis; moreover, its level is significantly and positively correlated with TC/LDL (86). IL-10, a key anti-inflammatory cytokine, improves insulin sensitivity by upregulating lipocalin; however, its levels are significantly reduced in RA patients, exacerbating metabolic imbalances (73, 77, 86).

RF and ACCP antibodies are involved in RA pathology through metabolic reprogramming. Elevated synovial G6PD activity in ACCP antibody-positive patients promoted the metabolic flow of the PPP, leading to the overproduction of NADPH and enhanced T-cell autoreactivity (84, 86, 87). RF can inhibit ABCA1-mediated reverse cholesterol transport and activate LOX-1 receptors to induce endothelial damage by forming RF-ox-LDL complexes. Moreover, ACCP antibody-positive patients had a 1.8-fold higher HOMA-IR index as compared to the negative group; single-cell sequencing revealed that their pancreatic islets had an increased infiltration of CD8+ T cells, which may be related to the molecular mimicry effect of citrullinated GAD65 (84). These findings provided new targets for intervention in the regulation of RA metabolism-immunity interactions (**Table 3**).

**Table 3:** Regulatory mechanisms and intervention strategies of glucose and lipid metabolism by key inflammatory markers.

Markers	Metabolic effects	Molecular mechanisms
TNF-α	Insulin resistance/hyperlipolysis	Inhibition of IRS-1 tyrosine phosphorylation and downregulation of GLUT4
IL-6	VLDL-TG abnormality/FAO inhibition	Activation of the JAK2/STAT3-RANKL axis and methylation of CPT1A
IL-1β	Hypercholesterolemia	Activation of the NLRP3-SCAP/SREBP2 axis and demethylation of the HMGCR promoter
Anti-CCP antibody	Enhanced metabolic flow of PPP	Upregulation of G6PD activity and imbalance of NADPH/ROS ratio
RF	Reverse cholesterol transport disorder	Formation of ox-LDL complexes and Inhibition of ABCA1 expression

## 6 Innovative targets and intervention strategies for the regulation of glycolipid metabolism in RA treatment

### 6.1 HK2 as a novel metabolic target for RA therapy

HK2, an initiating and rate-limiting enzyme of the glycolytic pathway, plays a central regulatory role in the metabolic reprogramming and invasive phenotype of RA FLS. In RA patients, HK2 is highly expressed in the synovium, and it inhibits apoptosis and promotes FLS proliferation by acting on mitochondrial channel VDAC (82, 83, 88). It can block the release of cytochrome c by binding to mitochondrial voltage-dependent anion channels (VDAC), inhibit FLS apoptosis, and promote its abnormal proliferation (82, 83). Meanwhile, the *HK2* gene silencing reduced FLS migration and invasiveness by 62% and 78%, respectively, while its overexpression significantly enhanced lactic acid production and synovial hyperplasia (88). HK2 activates the HIF-1α signaling axis, forming a ‘glycolysis-hypoxia’ positive feedback loop, which upregulates GLUT1/LDHA expression and drives pathological vascular opacification. Due to the low expression of HK2 in T cells, it has little effect on the systemic immune system, making it an ideal target with therapeutic selectivity (87, 88).

Significant progress has been made in targeted intervention strategies against HK2. The small molecule inhibitor 3-bromopyruvic acid (3-BrPA) could effectively attenuate synovial inflammation and bone erosion in a CIA model by inhibiting enzyme activity through alkylation of the ATP-binding domain of HK2 (82). Stimulator of interferon genes (STING) proteins can directly bind to and inhibit HK2 while promoting mitochondrial FAO, thus achieving a synergistic effect of metabolic reprogramming and anti-inflammation (2). WTAP-mediated modification of m6A methylation can enhance HK2 mRNA stability; siRNA therapy targeting this axis could significantly reduce DAS28 scores in an RA mouse model (83). Ketoconazole can induce phagocytosis of FLS by inhibiting the HK2 mitochondrial localization and reduce dose-dependent toxicity in combination with methotrexate (83, 86, 88).

Clinical translational research on HK2-targeted therapies has entered a new phase. 3-BrPA showed selective inhibition of FLS glycolytic activity in synovial biopsies from RA patients while reducing mitochondrial ROS levels by 45%, suggesting both metabolic regulation and oxidative stress mitigation effects (15). Genomic analysis revealed that the patients carrying the rs7604190 polymorphism in the *HK2* gene exhibited a 1.8-fold increase in response rate to tofacitib, a JAK inhibitor, suggesting the potential of metabolism-immunity cross-regulation for personalized therapy (15). The novel HK2-PET imaging technique can quantitatively assess synovial glycolytic activity using standardized uptake values (SUVs), and its strong correlation with DAS28 scores can provide a precise tool for efficacy monitoring. Future studies should deeply analyze the synergistic mechanisms of the HK2-lipid metabolism axis (such as SCAP-SREBP2 regulatory network) and explore its combined therapeutic strategies with biological agents (89) (**Table 4**).

**Table 4:** HK2 as a novel metabolic target for RA therapy.

Categories	Key Elements
Expression profile	HK2 was highly expressed in the synovial lining of RA (co-localized with CD90/CD55) while having low expression in OA.
Molecular mechanisms	1. Binding to VDAC blocks cytochrome c release and inhibits apoptosis 2. Activation of HIF-1a → up-regulation of GLUT1/LDHA → driving the “glycolysis-hypoxia” feedback → pannus formation
Effects on FLS function	Gene silencing: migration ↓62%, invasion ↓78%; Overexpression: lactate ↑, synovial thickening
Advantages of targeted therapy	Low T cell expression → little systemic immune effect
Inhibitors	3-BrPA: alkylated ATP-binding domain → Inhibitory activity → Inflammation/bone erosion in the CIA model ↓
Strategies for metabolic regulation	1. STING binding HK2→ glycolysis ↓+CPT1A↑ 2.1-fold (fatty acid oxidation ↑) 2. WTAP mediated m6A modification → HK2 mRNA stabilization → siRNA therapy reduced DAS28 score
Drug combination	Ketoconazole: inhibition of HK2-mitochondrial binding → autophagy ↑; in combination with methotrexate → Toxicity ↓

### 6.2 Novel strategies for lipid metabolism regulation in RA therapy

The regulation strategy of RA lipid metabolism has been developed into a multidimensional precision intervention system, focusing on metabolic nuclear receptor regulation as well as targeting key enzymes. The LXR agonist GW3965 could enhance macrophage cholesterol reverse transporter by upregulating ABCA1 expression, thereby significantly reducing synovial lipid accumulation and joint inflammation; however, it has pro-lipid-synthesizing side-effects, and the therapeutic efficacy can be optimized by selective targeting of the LXR β-subtype (15, 78, 88). The abnormal activation of choline kinase alpha (Chokα) in the synovial microenvironment catalyzes choline-PC metabolic conversion, thereby promoting FLS invasion and MMP expression. On the other hand, the specific inhibitor MN58b can significantly inhibit FLS migration capacity. PPARγ ligand rosiglitazone effectively reduced synovial TG synthesis and ameliorated metabolic inflammation by inhibiting SREBP-1c-mediated FASN expression, confirming the key role of the synergistic regulation of nuclear receptor-lipid synthase in reversing RA lipotoxicity (15, 88).

The interactive regulation of pro-inflammatory factors and lipid metabolism offers new dimensions for therapy. T cells from RA patients can inhibit mitochondrial FAO by activating the PI3K/Akt pathway, leading to abnormal accumulation of intracellular lipid droplets and enhanced Th17 differentiation; moreover, the metabolic imbalance can be reversed by the PI3K inhibitor LY294002 (2, 84). Malak Alannan et al. revealed that TNF-α promoted sphingomyelin hydrolysis to ceramide (Cer) by activating nSMase-2, thereby inducing FLS apoptosis resistance; on the other hand, the Cer analogue, C2-ceramide, could restore its sensitivity to apoptotic signaling (85). The natural flavonoid quercetin reduced the proliferative and invasive capacity of FLS by inhibiting the IL-21/RAS/Chokα signaling axis and simultaneously ameliorated lipid peroxidation damage. Furthermore, the FASN inhibitor TVB2640 induced endoplasmic reticulum stress in FLS by blocking palmitate synthesis, which produced a synergistic anti-inflammatory effect in combination with methotrexate (88).

Innovative strategies, targeting lipid metabolites, drive the development of personalized therapies. The S1P receptor modulator fingolimod could significantly reduce synovial thickening by inhibiting FLS migration and angiogenesis (88). The patients carrying polymorphisms in the *SREBP-1* gene are sensitive to the ACLY inhibitor SB-204990; the mechanism involves inhibition of acetyl coenzyme A production and epigenetic regulation (2, 15). CD36 monoclonal antibody could reverse the lipid-dependent phenotypes of T cells by blocking fatty acid uptake and enhancing the efficacy of TCR signaling, thus providing new ideas for metabolism-immunity synergistic intervention (89). These advances mark a new phase of multi-target, spatio-temporal specific modulation in RA therapy.

### 6.3 Promise of multi-targeted combination therapies in RA treatment

The core of multi-target combination therapy lies in remodeling the metabolic-immune homeostasis in the pathological microenvironment of RA through the synergistic metabolic intervention and immunomodulation. Studies have confirmed that the combination of glycolysis inhibitors and classical immunomodulators exhibits a significant synergistic effect. Through the dual blockade of the NF-κB/MAPK signaling pathway and purine metabolism, the combination of PFKFB3 inhibitor PFK15 and methotrexate (MTX) reduced IL-6/TNF-α levels by 48% as compared to the monotherapy (84, 89, 90). Anti-TNF-α biologics (such as adalimumab) in combination with 2-deoxyglucose (2-DG) synergistically restored abnormal T-cell lipid metabolism and mitochondrial FAO capacity, as well as inhibited Th17 differentiation. The combination of JAK inhibitor tofacitib and PFK15 restored ATP production to 75% of physiological levels by modulating the STAT3-HK2/LDHA axis and improved glycolytic activity and mitochondrial function simultaneously (78, 85, 86).

The combined intervention strategies targeting the glycolipid metabolic network might restore metabolic disturbances more comprehensively. HK2 inhibitor 3-BrPA in combination with LXR-β agonist GW3965 could effectively alleviate the synovial lipotoxic microenvironment by blocking glycolytic flux and activating ABCA1-mediated cholesterol efflux (91). PFKFB3 inhibitor PFK15 combined with SCD1 inhibitor CAY10566 could synergistically inhibit pro-inflammatory monounsaturated fatty acid synthesis in synovial fibroblasts and significantly reduce IL-1β and MMP-3 levels. The synergistic intervention strategies across metabolic pathways are equally effective. The combination of choline kinase-α inhibitor MN58b and AMPK agonist metformin could reduce synovial vascular opacification area by 43% through dual inhibition of phosphatidylcholine synthesis and glycolytic enzyme expression (23).

Current research has explored the combined application of HK2 inhibitors (e.g., 2-DG) and AMPK agonists (e.g., Metformin) in RA models, demonstrating synergistic inhibitory effects on synovitis and bone erosion. Meanwhile, LXR agonists and LDHA inhibitors have shown potential for coordinated regulation of lipid metabolism and glycolysis in animal experiments (23). However, most of the aforementioned combination therapies are still in preclinical stages. Currently, only Metformin has obtained extensive clinical approval as a treatment for type 2 diabetes, while its indication expansion for RA remains in exploratory trials (e.g., NCT05110044). LXR drugs have not yet received clinical approval due to concerns about side effects. Regarding patient stratification strategies, preliminary studies on metabolic phenotypes (e.g., PE/LPA concentration gradients and HK2 expression profiles) suggest that classifying RA patients by metabolic activity status could guide the selection of appropriate candidates for combination therapy. However, large-scale prospective studies remain scarce, necessitating the establishment of unified biomarker stratification criteria and regulatory pathways. The multi-target intervention strategy provides theoretical support for overcoming RA’s metabolic adaptability. Future combination therapies may break through the limitations of single-target treatments, thereby improving response rates and disease remission depth.

## 7 Summing up and looking forward

Rheumatoid arthritis (RA), a chronic autoimmune disease characterized by symmetrical polyarthritis, features glycolipid metabolism abnormalities as both a secondary inflammatory response and a core driver of disease progression. This vicious cycle is driven by metabolic reprogramming, amplified inflammatory signaling, and epigenetic regulation. Precision intervention strategies targeting key glycolytic enzymes, lipid metabolism nodes, and metabolic-immune interactions offer new avenues to disrupt this “metabolic-inflammatory” cycle. For RA patients with type 2 diabetes mellitus (T2DM), personalized metabolic interventions are particularly crucial. AMPK agonists like metformin not only improve insulin sensitivity but may also enhance anti-inflammatory effects through synergistic regulation of lipid synthesis and glycolytic activity. However, JAK inhibitors and HK2 inhibitors may exhibit altered pharmacokinetics and inflammatory target expression in diabetic contexts, requiring further validation of their efficacy and safety through multicenter clinical studies. Future therapeutic designs should address the heterogeneity of RA patients’ metabolic phenotypes, especially metabolic network remodeling in coexisting T2DM. Research needs to elucidate spatiotemporal-specific metabolic regulatory networks, develop dynamic assessment systems based on metabolomics, and explore personalized treatment strategies combining targeted metabolism and immune pathways to improve long-term prognosis and systemic complication management in RA patients.
