# Correction: Crossing the metabolic homeostasis divide: panoramic decoding of therapeutic targets for metabolic-inflammatory crosstalk in rheumatoid arthritis

**DOI:** 10.3389/fimmu.2025.1704527

**Published:** 2025-11-26

**Authors:** Siyu Liang, Lei Wan, Siyu Wang, Mengyu Zhang, Ying Wang, Wenwen Min, Yu Zhang

**Affiliations:** 1 The First Affiliated Hospital of Anhui University of Chinese Medicine, Anhui, Hefei, China; 2 Anhui University of Chinese Medicine First Clinical Medical College, Anhui, Hefei, China

**Keywords:** rheumatoid arthritis, glucose metabolism, lipid metabolism, inflammations, immunity, target of intervention

There was a mistake in **Figure 3** as published. During post-publication review, **Figures 2** and **3** were identified as requiring improvement for clarity and coherence. To provide a more concise and integrated visualization, both figures have been removed and replaced with a single updated and fully original figure. This modification does not affect the scientific conclusions of the article. The corrected **Figure 2** and its caption appears below.

**Figure 2:**
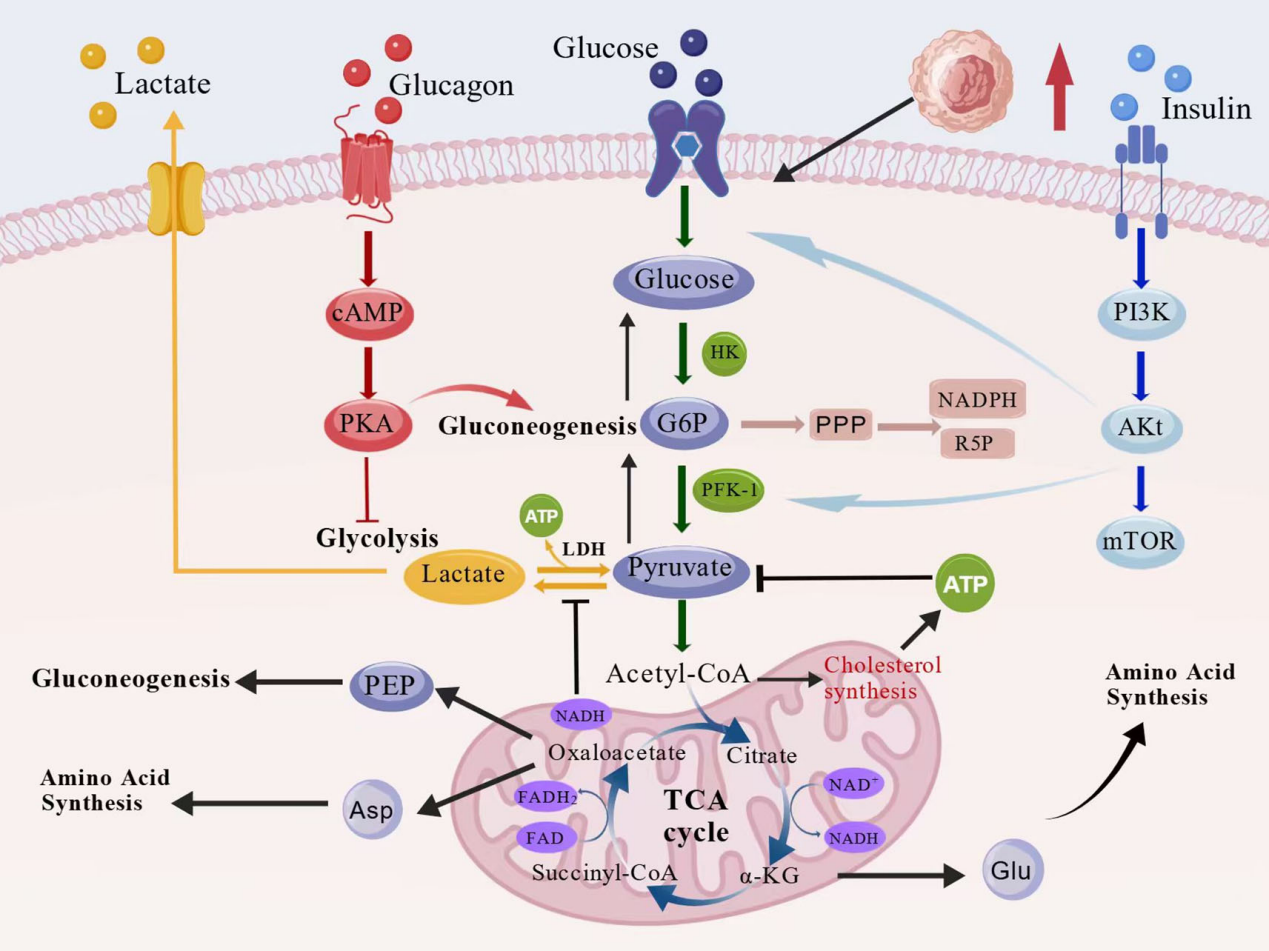
Core pathways of glucose metabolism and their dynamic synergistic networks. Glucose metabolism integrates glycolysis, the tricarboxylic acid (TCA) cycle, and amino acid and lipid synthesis. Insulin activates the PI3K–Akt–mTOR pathway to enhance glucose uptake and glycolysis, whereas glucagon promotes gluconeogenesis through the cAMP–PKA cascade. The TCA cycle acts as a metabolic hub linking carbohydrate, amino acid, and lipid metabolism through acetyl-CoA and key intermediates.

The original version of this article has been updated.

